# Acute Necrotizing Pancreatitis Following Intragastric Balloon Insertion

**DOI:** 10.7759/cureus.54437

**Published:** 2024-02-19

**Authors:** Siddharth Sankar Das, Walid Bondok, Iqra F Jafri, Dina A Ghazi, Zaid AbdelAziz

**Affiliations:** 1 General Surgery, Dubai Hospital, Dubai, ARE; 2 General Surgery, Dubai Medical College, Dubai, ARE; 3 Pediatrics, Tawam Hospital, Abu Dhabi, ARE

**Keywords:** pancreatitis causes, bariatric case report, weight loss and obesity, gastric balloon, necrotising pancreatitis

## Abstract

Obesity has become a widespread global issue, particularly in the developed world. One popular weight loss technique is the intragastric balloon placement due to its simplicity of insertion and safe nature. While some side effects have been linked to its use, most are benign. However, severe complications do occur in some cases. One such rare complication is pancreatitis due to compression of the pancreas or the pancreatic duct. We encountered an interesting case of necrotizing pancreatitis following gastric balloon insertion, about which scarce data is available in the literature; its incidence is also unknown currently. Our patient was a 22-year-old male with a gastric balloon inserted for obesity eight months before his presentation. The mechanism of the inflammation, the age of the patient, and the progression to necrosis are the compelling aspects of this case.

## Introduction

Many noninvasive and invasive weight loss techniques have been introduced to combat the obesity pandemic. Of these, a popular minimally invasive method is the intragastric balloon placement. Although the weight loss gained by this procedure is typically not significant, it is associated with relatively few side effects and complications, and it tends to be well tolerated by most people. While an intragastric balloon is usually kept in place for up to six months, balloons that may remain in the stomach for up to 12 months are also available. The expected weight loss from this procedure is around 15-20%. However, this varies considerably between different types of balloons [1,2]. This procedure is favored by a large segment of the obese population to aid in their efforts to lose more weight.

Common complications of intragastric balloon insertion tend to be mild, such as gastric ulcers, erosions, and reflux esophagitis. The incidence of these mild complications is quite low (less than 2% of patients experience esophagitis and less than 1% have gastric erosions; however, these values vary for different types of balloons) and the complications are managed conservatively in most cases, leaving the balloon in situ. Very few serious complications such as gastric, esophageal, or balloon rupture have been reported, and these are extremely rare; therefore, it is seen as a relatively safe procedure [3-5]. This case report is unique as it discusses a rare severe complication in a patient who underwent intragastric balloon insertion. We present a case of a patient with acute necrotizing pancreatitis due to compression of the pancreas by an intragastric balloon, a complication that is rarely reported in the literature.

## Case presentation

Our patient was a 22-year-old male who underwent an intragastric balloon insertion to reduce weight eight months before his acute presentation. His BMI had been 45.63 kg/m^2^ and he had no other comorbidities. He had experienced no other complaints due to the gastric balloon until his presentation to the hospital, and he had lost 22 kilograms of weight until then, reducing his BMI to 38.73 kg/m^2^.

He presented to the hospital with a one-day history of severe upper abdominal pain, which was associated with food intake. No history of vomiting or change in bowel habits was reported, and he had no other systemic symptoms. The patient's surgical and medical history was otherwise unremarkable, and he denied alcohol consumption or any medication use. The patient had a blood pressure of 163/89 mmHg on examination but was otherwise vitally stable. The abdominal examination revealed severe tenderness in the upper abdomen, while the rest was soft. The systematic examination was unremarkable. His laboratory studies were significant for elevated white blood cells and raised amylase and lipase levels (Table 1).

**Table 1:** Laboratory values on admission

Laboratory investigation	Result	Normal value
Complete blood count		
White blood cells	14.2	3.6-11 x 10^9^/L
Hemoglobin	13	13-17 g/dL
Hematocrit	39.8	40-50%
Platelets	328	150-410 x 10^9^/L
Pancreatic enzymes		
Serum amylase	379	28-100 U/L
Serum lipase	642	13-60 U/L
Inflammatory markers		
C-reactive protein	24.8	<5 mg/L
Procalcitonin	0.10	<0.05 ng/ml
Liver function test		
Total bilirubin	0.2	0.1-1.2 mg/dL
Alkaline phosphatase	87	40-129 U/L
Alanine transaminase	88	5-30 U/L
Lactate dehydrogenase	262	140-280 U/L
Lipid profile		
Triglyceride	107	<150 mg/dL
Total cholesterol	106	<200 mg/dL
Low-density Lipoproteins	45	<115 mg/dL
Calcium	9.7	8.9-10.2 mg/dL

**Figure 1:**
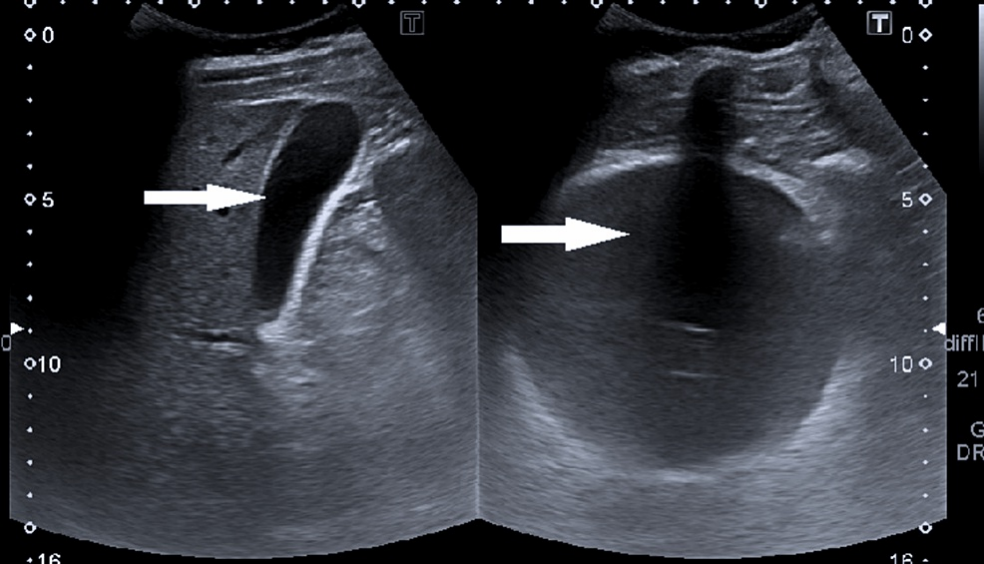
Ultrasound of the abdomen showing an intragastric balloon

**Figure 2:**
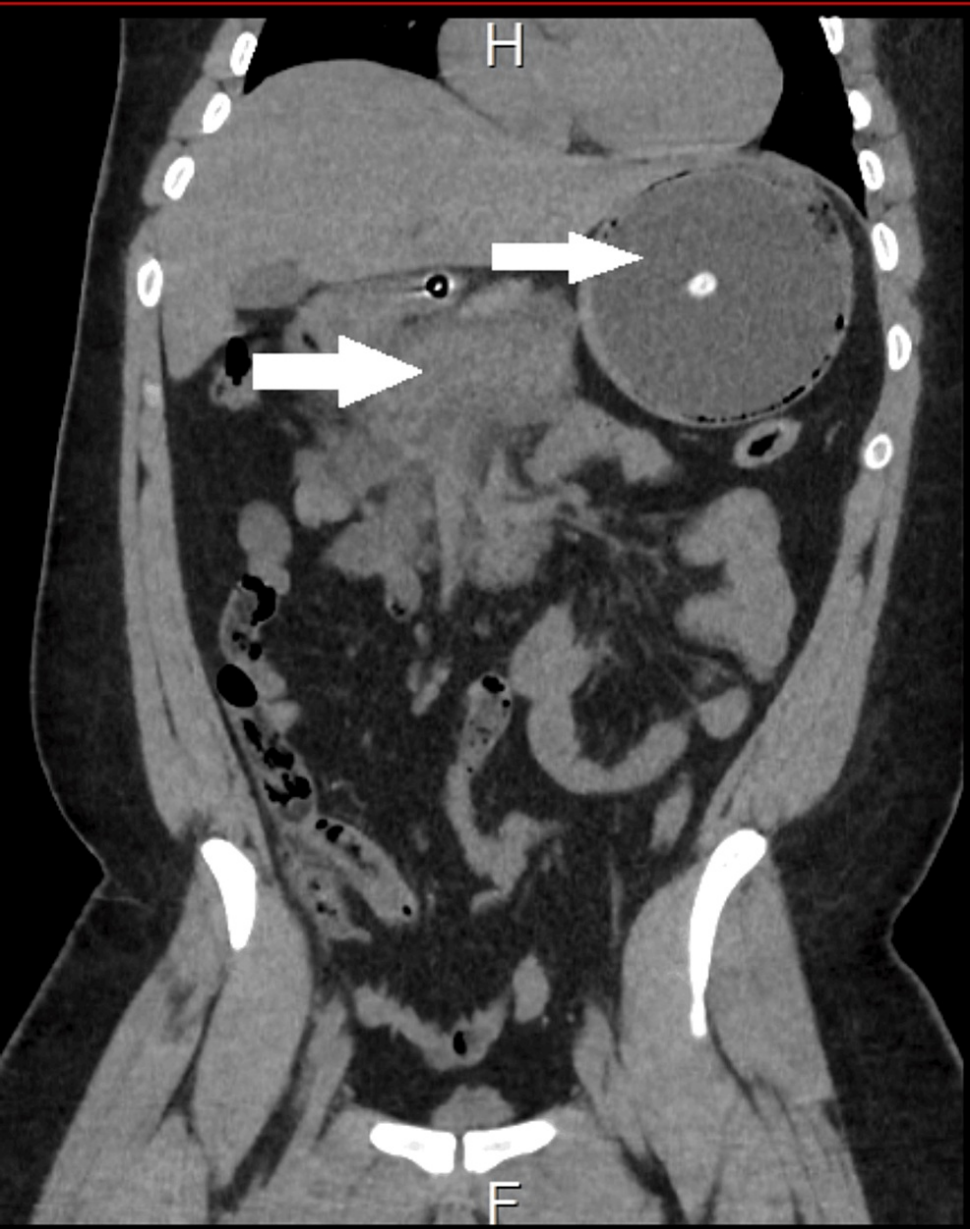
CT scan of the abdomen showing an intragastric balloon with acute pancreatitis and peripancreatic fluid collection CT: computed tomography

An initial abdominal ultrasound revealed a well-inflated intragastric balloon and a poorly visualized pancreas (Figure 1). Therefore, the study was followed up with a CT scan of the abdomen with contrast, which showed extensive peripancreatic collection and fat plane inflammatory stranding, suggestive of acute pancreatitis (Figure 2). The patient then underwent a magnetic resonance cholangiopancreatography (MRCP) the following day, which confirmed the diagnosis of acute interstitial edematous pancreatitis due to a non-biliary cause, as the common bile duct was not dilated, and the gallbladder appeared normal. 

**Figure 3:**
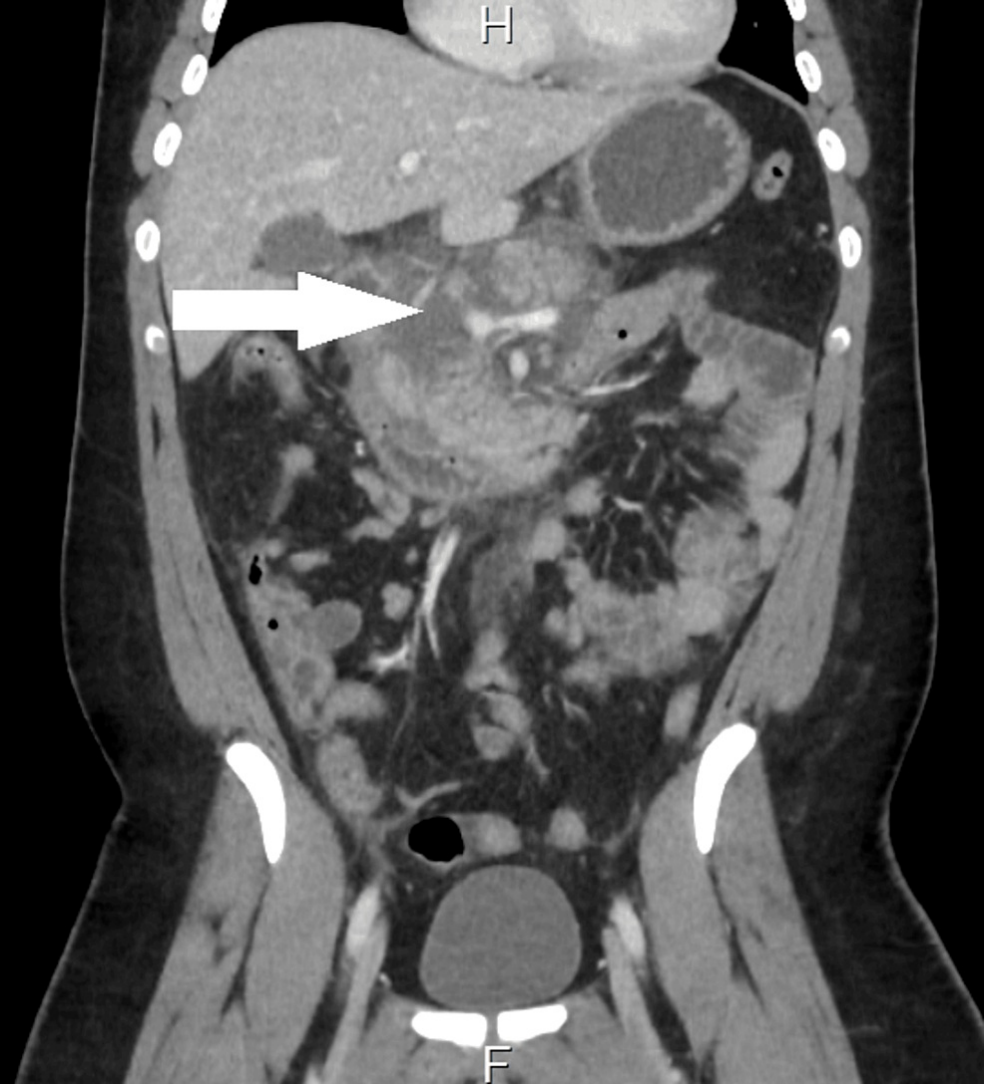
CT scan of the abdomen after removal of the intragastric balloon showing progression of pancreatitis with pancreatic necrosis CT: computed tomography

The patient was admitted as a case of non-biliary pancreatitis. The gastric balloon was removed as it was suspected to be the cause of the inflammation and necrosis of the pancreas, based on a lack of any other risk factors and some case reports describing similar complications from other parts of the world. A follow-up CT with contrast two days later showed the swollen, edematous, and necrotic appearance of a pancreatic head and body with surrounding fat smudging and creeping peripancreatic, retroperitoneal, and perihepatic fluid signals (Figure 3). After the removal of the intragastric balloon, the patient was managed conservatively with intravenous hydration; analgesia, and proton pump inhibitors. He was discharged from the hospital following improvement of his symptoms.

**Figure 4:**
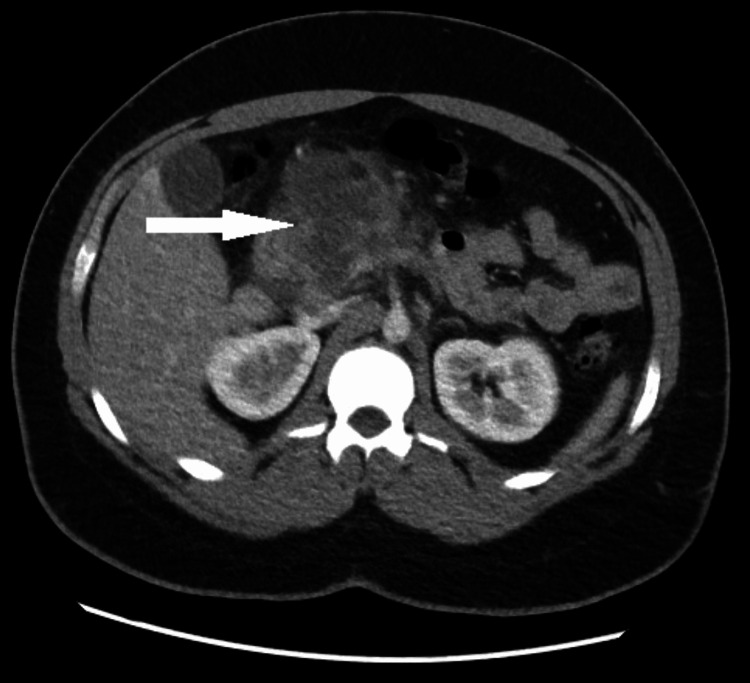
CT scan of the abdomen showing extensive necrosis of the head of the pancreas with peripancreatic fluid collection CT: computed tomography

However, the patient again presented to the emergency with a similar clinical picture 12 days later. An ultrasound was inconclusive due to overlying bowel gasses; however, an abdominal CT scan with contrast showed an edematous pancreas with necrosis of the pancreatic head and multiloculated prepancreatic fluid collection, denoting a pancreatic pseudocyst (Figure 4).

He was managed conservatively with intravenous fluids, adequate analgesia, antibiotics, and proton pump inhibitors. The patient's symptoms improved, and he was discharged seven days later with an appointment for MRCP. He followed up with an MRCP as an outpatient, which showed extensive irregularly shaped collections in the pancreas and adjacent extra-pancreatic tissue. The pancreatic duct was found to be narrowed near the collection, probably due to post-inflammatory stenosis or spasm. The patient was followed up and he subsequently recovered without any lasting consequences of the infection.

## Discussion

Obesity is rapidly becoming a global pandemic, with the WHO reporting a tripling in the number of obese individuals since 1975 [6]. This has resulted in several health-related issues due to obesity-related problems such as osteoarthritis, metabolic disease, and coronary artery disease, to name a few [7]. With increasing global awareness of the adverse effects of obesity, many weight reduction methods have been introduced. To achieve quick and effective results, invasive surgical procedures and other less invasive methods, including intragastric balloon insertion, are becoming popular. Although bariatric surgery has been a popular method due to the much more significant reduction in weight as well as its noninvasive and safe nature, intragastric balloon insertion is also a widely favored method. Some common side effects of the intragastric balloon are nausea, the feeling of satiety, and gastric erosions. More severe side effects, such as obstruction or perforation, are infrequent [8].

Since the use of intragastric balloons has become widespread, fewer than 40 cases of acute pancreatitis directly attributed to balloon insertion have been reported. It is theorized that pancreatitis is caused by pressure from the balloon on the duct or body of the pancreas. However, the specific pathological sequence is not fully elucidated yet. None of the reported cases had necrosis in the pancreas. While many of these cases were managed conservatively without the removal of the balloon, some of them required the removal of the balloon with or without other management strategies, along with standard inpatient management of acute pancreatitis. The prognosis has been generally excellent in all reported cases [9-17].

Acute pancreatitis commonly presents in the third to sixth decade of life [18], while reported cases of pancreatitis due to intragastric balloon insertion tend to feature younger patients. The most common causes of acute pancreatitis are alcohol use and gallstones. Other less common causes include hypertriglyceridemia and anemia, with scorpion stings being a classically rare cause [19]. Necrotizing pancreatitis presents similarly to acute pancreatitis and the two conditions are associated with similar risk factors. Necrotizing pancreatitis accounts for around 20% of all pancreatitis cases presenting to the emergency department. On CT with contrast, necrotizing pancreatitis is characterized by the lack of enhancement of a large proportion of the pancreas [20].

Our patient, although young, presented with classical signs and symptoms of acute pancreatitis. Given his lack of other risk factors and the recent insertion of an intragastric balloon for weight loss and improvement of his symptoms on the removal of the balloon, it is likely that the cause of the pancreatitis was the intragastric balloon itself. The dangerous nature of necrotizing pancreatitis necessitates a great deal of care in including such diagnoses in the differential. Our prime objective in reporting this case is to highlight a rare complication of intragastric balloon insertion, especially because no previous cases of acute pancreatitis due to an intragastric balloon have been reported in our city. Although there have been a few cases of pancreatitis post-intragastric balloon insertion in other parts of the world, to our knowledge, there have been no other reports of pancreatic necrosis attributed to this procedure.

## Conclusions

Clinicians need to be aware of the common as well as the rare-but-severe complications of any procedure. This also applies to relatively safe procedures such as intragastric balloon insertion. Also, emergency physicians and surgeons need to suspect such complications in seemingly young and healthy patients if they have a history of weight loss procedures and interventions, even in the absence of other risk factors, to prevent long-term adverse effects. Patients undergoing such procedures should be counseled regarding the possible risks pre-procedure and offered other options available for weight loss.

Our study highlights the importance of maintaining a high index of suspicion for pancreatitis in patients post intragastric balloon insertion and brings to light the risks associated with this complication, as necrotizing pancreatitis requires swift and prompt management.
